# Human papillomavirus type 58: the unique role in cervical cancers in East Asia

**DOI:** 10.1186/2045-3701-2-17

**Published:** 2012-05-09

**Authors:** Paul KS Chan

**Affiliations:** 1 Department of Microbiology, The Chinese University of Hong Kong, 1/F Clinical Sciences Building, Prince of Wales Hospital, Shatin, New Territories, Hong Kong Special Administrative Region, People’s Republic of China

## Abstract

**Background:**

About 15 types of human papillomavirus (HPV) are classified as high-risk based on their epidemiological link with cervical cancer. These HPV types have deferent degrees of oncogenicity and their distribution among cervical precancers and cancers varies ethnogeographically. HPV58 is rare worldwide but being found more commonly in East Asia.

**Findings:**

A high prevalence of HPV58 among squamous cell carcinoma has been reported from China (28% in Shanghai, 10% in Hong Kong and 10% in Taiwan) and other countries in East Asia including Korea (16%) and Japan (8%). HPV58 ranks the third in Asia overall, but contributes to only 3.3% of cervical cancers globally. The reasons for a difference in disease attribution may lie on the host as well as the virus itself. HLA-DQB1*06 was found to associate with a higher risk of developing HPV58-positive cervical neoplasia in Hong Kong women, but not neoplasia caused by other HPV types. An HPV58 variant (E7 T20I, G63S) commonly detected in Hong Kong was found to confer a 6.9-fold higher risk of developing cervical cancer compared to other variants. A study involving 15 countries/cities has shown a predilection in the distribution of HPV58 variant lineages. Sublineage A1, the prototype derived from a cancer patient in Japan, was rare worldwide except in Asia.

**Conclusions:**

HPV58 accounts for a larger share of disease burden in East Asia, which may be a result of differences in host genetics as well as the oncogenicity of circulating variants. These unique characteristics of HPV58 should be considered in the development of next generation vaccines and diagnostic assays.

## Disease burden of cervical cancer

Human papillomavirus (HPV) plays a necessary, though insufficient, role in the development of cervical cancer, which is the third most common cancer in women worldwide, just following breast and colorectal cancers [1,2]. It has been estimated that about 530 000 new cases and 275 000 deaths from the disease occurred in 2008. The incidence of cervical cancer varies dramatically across the world, which is mainly related to the availability and accessibility of cervical screening programs. Most places in South America and South and West Africa have an age-standardized incidence above 20 per 100 000 women per year, and some places in these regions have reached 40 per 100 000 women per year. In contrast, the age-standardized incidence rates were below 10 per 100 000 women per year in North America, Western Europe, Australia and New Zealand. Even within Asia, the age-standardized incidence also varies substantially with 9.6 per 100 000 women per year in East Asia, 15.8 per 100 000 women per year in South-Eastern Asia, 24.6 per 100 000 women per year in South-Central Asia and 4.5 per 100 000 women per year in Western Asia [2].

## HPV and cervical cancer

Papillomaviruses have a small double-stranded DNA genome of about 8 kb long. To date, more than 120 types of HPV have been well characterized, of which about 40 types can infect the genital tract [3]. About 15 types of these genital (mucosal) HPV are classified as “high-risk” because of their oncogenic or possible oncogenic properties either demonstrated by in-vitro biochemical studies or inferred from epidemiological observations [4,5]. Two early proteins, E6 and E7, are the main oncoproteins encoded by high-risk HPV [6,7]. E6 protein binds to the tumour suppressor protein p53 in associate with the E6-associated protein (E6-AP). Overexpression of E6 results in the degradation of p53, anti-apoptosis, chromosomal destabilization, enhancement of foreign DNA integration and activation of telomerase. E7 binds to retinoblastoma protein (Rb) and Rb-related pocket proteins resulting in inactivation of Rb-related pocket proteins, activation of cyclins, inhibition of cyclin-dependent kinase inhibitors, and enhancement of foreign DNA integration and mutagenesis.

## Distribution of HPV types

HPV16, 18, 31, 33, 35, 39, 45, 51, 52, 56, 58 and 59 are regarded as high-risk types [4,8]. HPV16 and HPV18 contribute to most cervical cancers, accounting respectively for about 59% and 13% of squamous cell carcinoma, and 36% and 37% of adeno/adenosquamous carcinoma worldwide [9]. While there is little variation in the prevalence of HPV16 and HPV18 among cervical cancers across the world, the contribution of other types varies geographically. The currently available prophylactic vaccines target two high-risk types, HPV16 and HPV18. The efficacy of these vaccines is mainly type-specific, although some cross-type protection has been observed especially for the bi-valent vaccine (Cervarix**®**, GlaxoSmithKline Biologicals) [10]. Therefore, variation in the distribution of non-vaccine (non-HPV16/18) types would have an implication on the design of next generation vaccines.

## HPV58 in East Asia

Since mid 1990s, a few studies have reported a higher prevalence of HPV58 among cervical squamous cell carcinoma cases in the southern and eastern parts of China [11-13]. With the increase in availability of commercial assays for the identification of multiple types of HPV, and the increase in demands on HPV type distribution data for assessing vaccine cost-effectiveness; a large amount of data have been generated during the last decade. At present, there is a strong body of evidence to show that HPV58 attributes to a substantial proportion of cervical cancers in the eastern and southern parts of China. For instance, HPV58 was found in 26% of squamous cell carcinoma in Shanghai [12], 10% in Hong Kong [14] and 10% in Taiwan [15]. These prevalence rates are much higher than those reported from other parts of the world [9]. The high prevalence of HPV58 is not limited to Chinese women, but also observed in the ethnogeographically related populations, particularly Korea (16%) and Japan (8%) [16,17]. HPV58 ranks the third among cervical cancer cases from Asia overall [18], but only contributes to 3.3% globally [19]. Furthermore, HPV58 has also been found in a relatively higher proportion of precancerous lesions (overall in 17.2% of cervical intraepithelial neoplasia grade 2/3) in East Asia [20]. Therefore, monitoring the prevalence of HPV58 in cervical precancers and invasive cancers in East Asia would provide an early indicator of HPV type replacement, if it ever occurs following the widespread administration of HPV16/18 vaccines.

## Reasons for higher prevalence of HPV58 in East Asia

The reason for a higher prevalence of HPV58 in East Asia is still not fully understood. The development of a clone of tumour cells from an HPV-infected epithelium is a multi-step process involving multiple factors in which host genetics is likely to be a main determinant. Our previous study on Hong Kong women has observed a positive association between HLA-DQB1*06 and HPV58-positive cervical intraepithelial neoplasia III/invasive cancers [21]. Of note, this risk association was type-specific and was not observed for cancers caused by other HPV types.

We have also observed that an HPV58 variant commonly found in Hong Kong was epidemiologically associated with a higher oncogenic risk [22]. This variant harboring two amino acid substitutions, T20I and G63S, in the E7 protein was found to associate with an odds ratio of 10.14 (95% confidence interval = 10.14–74.72) for the development of cervical cancer, which was 6.9-fold higher than HPV58 variants without these sequence variations. The first amino acid substitution (T20I) is close to the Leu-Xaa-Cys-Xaa-Glu domain that mediates association with the retinoblastoma protein (pRb) and its related proteins, p107 and p130. The substitution G63S results in a serine which could be phosphorylated by casein kinase II, and a positive association between phosphorylation rate and oncogenic potential of E7 has been reported [23].

## Lineage classification and geographical distribution

While studies from Hong Kong suggest that the host genetic factors and oncogenicity of circulating variants may play a critical role in the screwed ethnogeographical distribution of HPV58, further large-scale studies incorporating data from places with high and low prevalence of HPV58 are crucial to verify the observation and further explore the association. In this regard, it is instrumental to have a universally accepted classification system for HPV58 variants. Recently, two independent studies have reached the same conclusion and set an essential backbone for future studies [24,25]. The consensus is to classify HPV58 variants into four lineages designated as A (sublineages A1 and A2), B (sublineages B1 and B2), C and D (sublineages D1 and D2). Based on 401 isolates collected from 15 countries/cities across the four continents, lineage A was found to be the most prevalent across all regions [24]. Lineage C was found to be more frequent in Africa than elsewhere, whereas lineage D was more prevalent in Africa than in Asia. Of note, sublineage A1 which represents the prototype derived from a Japanese patient with cancer was rarely found worldwide, except in Asia. It is worthwhile to further investigate whether the reported higher contribution of HPV58 to invasive cancers in East Asia is due to a higher oncogenicity of sublineage A1. The study has also identified sequence signatures representing these lineages [24], allowing the conduction of large–scale molecular epidemiological studies on HPV58.

## Conclusions

The disease impact attributed to HPV58 varies ethnogeographically. The prevalence of HPV58 among cervical cancers found in East Asia is much higher than that reported from other parts of the world. This could be a result of differences in host genetic background as well as the circulation of HPV58 variants with different oncogenicity. These unique characteristics of HPV58 should be considered in the development of next generation vaccines and diagnostic assays.

## Competing interests

The author declared that he has no competing interests.
